# Association between newborn screening analytes and hypoxic ischemic encephalopathy

**DOI:** 10.1038/s41598-019-51919-x

**Published:** 2019-10-31

**Authors:** Lindsay A. Wilson, Deshayne B. Fell, Steven Hawken, Coralie A. Wong, Malia S. Q. Murphy, Julian Little, Beth K. Potter, Mark Walker, Thierry Lacaze-Masmonteil, Sandra Juul, Pranesh Chakraborty, Kumanan Wilson

**Affiliations:** 1 Clinical Epidemiology Program, Ottawa Hospital Research Institute, Ottawa Ontario, Canada; 2 School of Epidemiology and Public Health, University of Ottawa, Ottawa Ontario, Canada; 3 Children’s Hospital of Eastern Ontario Research Institute, Ottawa Ontario, Canada; 4 ICES, University of Ottawa, Ottawa Ontario, Canada; 5 Department of Obstetrics and Gynecology, University of Ottawa, Ottawa Ontario, Canada; 6 Department of Pediatrics, Cumming School of Medicine, University of Calgary, Calgary Alberta, Canada; 7 Department of Pediatrics, University of Washington, Seattle Washington, USA; 8 Department of Pediatrics, University of Ottawa, Ottawa Ontario, Canada; 9 Newborn Screening Ontario, Children’s Hospital of Eastern Ontario, Ottawa Ontario, Canada

**Keywords:** Biomarkers, Medical research

## Abstract

Hypoxic ischemic encephalopathy (HIE) is a major cause of neonatal mortality and morbidity. Our study sought to examine whether patterns of newborn screening analytes differed between infants with and without neonatal HIE in order to identify opportunities for potential use of these analytes for diagnosis in routine clinical practice. We linked a population-based newborn screening registry with health databases to identify cases of HIE among term infants (≥37 weeks’ gestation) in Ontario from 2010–2015. Correlations between HIE and screening analytes were examined using multivariable logistic regression models containing clinical factors and individual screening analytes (acyl-carnitines, amino acids, fetal-to-adult hemoglobin ratio, endocrine markers, and enzymes). Among 731,841 term infants, 3,010 were diagnosed with HIE during the neonatal period. Multivariable models indicated that clinical variables alone or in combination with hemoglobin values were not associated with HIE diagnosis. Although the model was improved after adding acyl-carnitines and amino acids, the ability of the model to identify infants with HIE was moderate. Our findings indicate that analytes associated with catabolic stress were altered in infants with HIE; however, future research is required to determine whether amino acid and acyl-carnitine profiles could hold clinical utility in the early diagnosis or clinical management of HIE. In particular, further research should examine whether cord blood analyses can be used to identify HIE within a clinically useful timeframe or to guide treatment and predict long-term health outcomes.

## Introduction

Hypoxic Ischemic Encephalopathy (HIE) is a condition in which inadequate oxygenation and blood flow result in clinical manifestations of brain dysfunction^1,2^, including seizures and issues with respiration^3^. A major cause of neonatal mortality and morbidity, HIE can result in developmental delays, epilepsy, cerebral palsy, and death^3–7^. Children who do not develop major disabilities are at increased risk for long-term sequelae including intellectual, verbal, and motor challenges^8^. HIE is estimated to affect approximately 1–6 infants per 1,000 live births in high-income countries^9^, but the incidence is higher in low- and middle-income countries, affecting approximately 20 infants per 1,000 live births and accounting for approximately one million deaths annually^10^. Some of the variability in the documented incidence of HIE in high-income countries, however, is partly due to the lack of a universally-accepted definition^3^.

A diagnosis of HIE is typically based on a combination of medical history, physical examination and umbilical cord gas analysis immediately after birth. Generally, one or more of the following signs must be present after birth: 10-minute Apgar score <5; need for prolonged resuscitation (>10 minutes); presence of meconium stained fluid; abnormal heart tracings; umbilical cord or first neonatal blood gas pH < 7.0, and/or base deficit >15 mmol/L^11^. The Sarnat staging criteria to establish severity of encephalopathy based on level of consciousness, muscle tone and reflexes, and autonomic function is also commonly used^12^. Untreated, one third of infants with the diagnosis of moderate or severe HIE have normal outcomes, and with therapeutic hypothermia, approximately half have normal outcomes. In addition, evidence suggests that a significant proportion of children thought to have mild HIE also exhibit signs of brain injury on MRI and may benefit from therapeutic hypothermia^13^. Thus, better diagnostic and prognostic biomarkers are needed to ensure correct selection of infants to treat and improved prognostication for parents.

Metabolic profiling of infants diagnosed with HIE has identified a number of putative biomarkers that predict injury severity and long-term outcomes, revealing several markers consistent with perturbations in energy metabolism associated with the condition^14^. These include altered Krebs cycle and amino acid metabolites, although these have not been validated nor demonstrated as feasible for translation into clinical practice^14^. Population-wide newborn screening to identify infants with rare, treatable congenital conditions involves analysis of newborn dried blood spots for several panels of analytes including amino acids, acyl-carnitines, hemoglobin variants, and enzymatic and endocrine markers. Routine newborn screening is available in most high-resource countries, although the array of markers analyzed by screening programs varies by jurisdiction. We hypothesized that routinely-collected newborn screening profiles could be used to identify a unique metabolic signature for HIE which would have the potential to guide diagnosis and care. In order to inform this potential opportunity for the development of a novel approach to identifying HIE in a clinical setting, the primary objective of our study was to assess whether there were any associations between the metabolic profiles generated through a provincial newborn screening program in Ontario, Canada and a diagnosis of HIE in a large, population-based cohort of infants.

## Results

### Demographics

**Figure 1:**
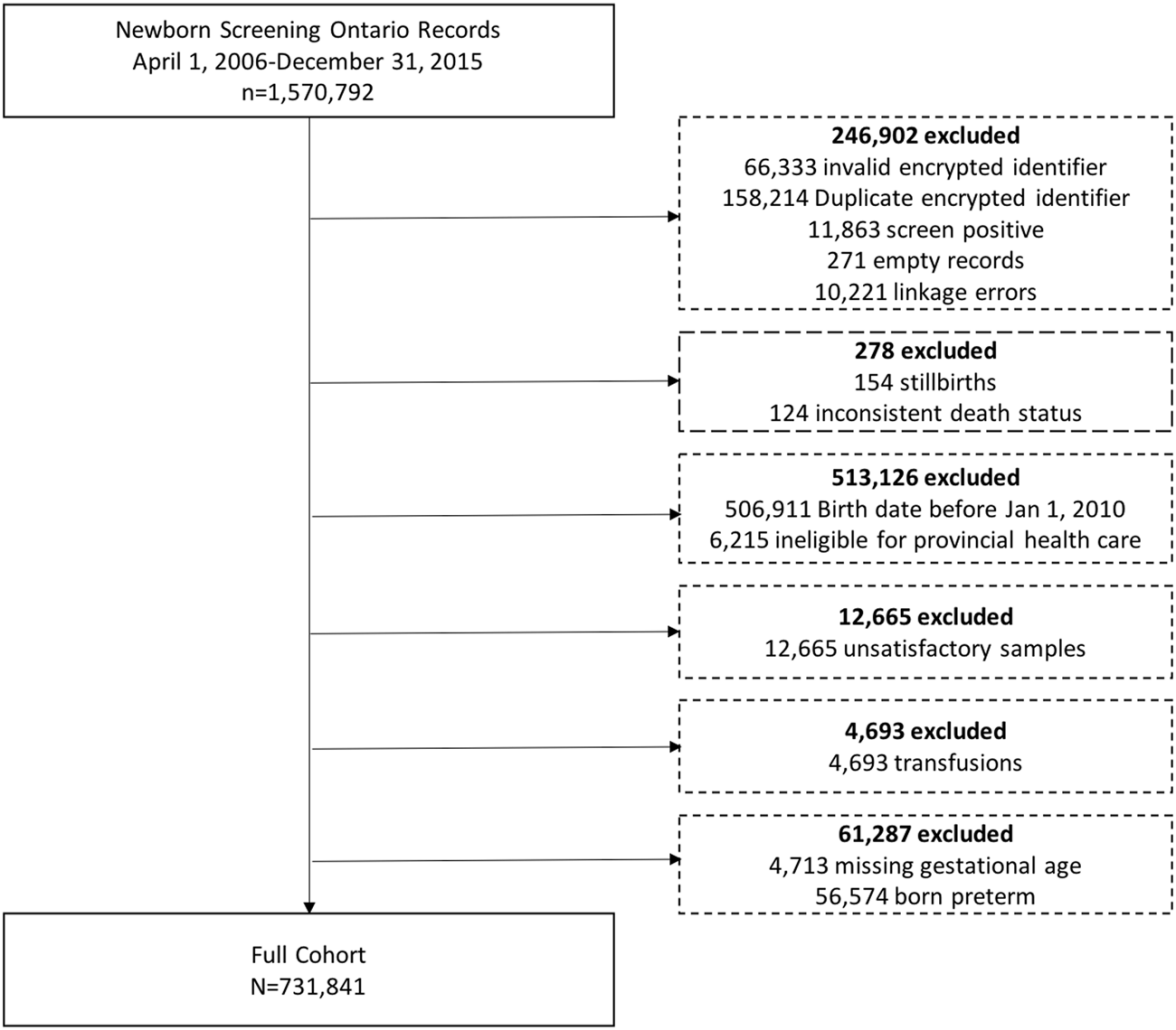
Flowchart of study exclusions.

After applying exclusion criteria, our cohort contained 731,841 term infants (Fig. 1), of whom 3,010 had a documented diagnosis of HIE during the neonatal period (4.11 per 1,000 term infants). Subject characteristics are provided in Table 1. HIE was more common among infants who were male (4.56 per 1,000 infants), those with a birthweight under 2,500 grams (6.39 per 1,000 infants), and those from a multifetal gestation (4.66 per 1,000 infants). Infants diagnosed with HIE more commonly received total parenteral nutrition (6% among infants with HIE vs. 0.2% among all infants) and had their newborn screening sample collected later than among the full study population (median 38 hours for infants with HIE vs. 28 hours among the overall cohort). Neonatal death also occurred more frequently among infants diagnosed with HIE compared with the full study population.

**Table 1:** Characteristics of study subjects.

Characteristic	Full study population n (%)^a^	Cases of HIE n (%)^a^	Rate of HIE per 1,000 infants
Total	731,841 (100)	3,010 (100)	4.11
Female	358,838 (49.03)	1,309 (43.49)	3.65
Male	372,997 (50.97)	1,701 (56.51)	4.56
Gestational age (completed weeks; range: 37–42 weeks): Mean ± SD	39.20 ± 1.15	39.44 ± 1.21	—
Birthweight (grams, range: 750–5155 g): Mean ± SD	3,426 ± 477	3,413 ± 528	—
<2,500	15,972 (2.18)	102 (3.39)	6.39
≥2,500	714,784 (97.72)	2,907 (96.61)	4.07
Multiple birth	13,533 (1.85)	63 (2.09)	4.66
Age at blood spot collection (hours, range: 0–7,242): Median (IQR)	27.87 (24.6–40.9)	38.83 (26.8–54.8)	—
Any total parenteral nutrition	1,301 (0.18)	181 (6.01)	—
Neonatal death	149 (0.02)	32 (1.06)	—

^a^Column percentage unless otherwise indicated.

### Correlation analysis

The five strongest partial correlations between analytes or analyte ratios and HIE during the neonatal period all contained amino acids. In particular, these were tyrosine, tyrosine:methionine, alanine:tyrosine, tyrosine:valine, and tyrosine:glycine. The full results of our correlation analysis are presented in the Supplementary Materials.

### Model performance

In order to examine the relationships between newborn screening analytes and HIE, we generated four distinct models. The results of this modeling can be found in Table 2. The first model comprised only the baseline clinical covariates specified in the Methods section. The adjusted c-statistic for this model was 0.611. The addition of relative fetal-to-adult hemoglobin levels in Model 2 did not affect the model performance, nor did the addition of 17-OHP and TSH (Model 3). The inclusion of the amino acids and acyl-carnitines in Model 4 resulted in improvement in model fit compared with the previous nested models (from 0.62 in Model 3 to 0.75 in Model 4, suggesting moderate performance).

**Table 2:** Model performance comparing baseline clinical model (Model 1) and clinical model plus newborn screening analytes for prediction of HIE.

Model		c- statistic^a,b^	c-statistic adjusted^a,b^	Optimism correction^a,b^	AIC (lower is better)	IDI^c^ (95% CI)	NRI^d^ (95% CI)
Model 1^e^	Infant sex, gestational age, birth weight, plurality, TPN	0.61225	0.6114	0.00085	37818	—	—
Model 2	Model 1 variables + fetal-to-adult Hb ratio	0.6135	0.61255	0.00095	37814	−0.000023 (−0.0001,0)	0.010117 (−0.0253, 0.0455)
Model 3	Model 2 variables + 17-OHP + TSH	0.6213	0.6194	0.0019	37738	0.000089 (0.0001, 0.0003)	0.045425 (0.0101, 0.0808)
Model 4^f^	Model 3 variables + restricted cubic spline terms for top five ranked analytes/analyte ratios + remaining analytes/analyte ratios	0.7477	0.7359	0.01185	34315	0.031953 (0.028, 0.0359)	0.65118 (0.616, 0.6864)

^a^C-statistic is equivalent to the area under the curve (AUC).

^b^Adjusted c-statistic is based on internal validation results using 200 bootstrap samples.

^c^Integrated Discrimination Improvement (IDI) quantifies the impact of additional variables on the average sensitivity of the preceding nested model (e.g., the IDI shown for Model 2 compares Model 2 with Model 1, etc.).

^d^Net Reclassification Improvement (NRI) quantifies the net increase/decrease in predicted values for the outcome compared to the preceding nested model (e.g., the NRI shown for Model 2 compares Model 2 with Model 1, etc.).

^e^Baseline model containing only clinical variables.

^f^Final fitted model.

Table 2 further illustrates the improvement of Model 4 over the other models. Specifically, Model 4 demonstrates better fit than the other models (AIC = 34315 in Model 4 compared to 37818 in the baseline model) even when factoring for the increased complexity of Model 4. The final model also showed improvement over the preceding models in terms of the predicted probabilities between events and non-events (IDI = 0.03), signifying better discrimination of HIE cases from non-cases. Lastly, misclassification was substantially reduced in Model 4, with over 65% of cases and non-cases who were initially misclassified being correctly reclassified (NRI = 0.65).

## Discussion

Our study has demonstrated a moderate association between patterns of metabolites identified through routinely collected newborn screening samples and a diagnosis of HIE. In particular, levels of amino acids and acyl-carnitines (and ratios of these analytes, most notably ratios including tyrosine) improved the ability of statistical models to identify infants with HIE compared to models derived from clinical variables alone. Adjusted c-statistics for these models demonstrated moderate fit. Our results and those of other groups increasingly indicate that newborn screening profiles can be used to identify infants at risk of developing serious perinatal health conditions, including HIE and death^15^. These findings have the potential to inform future research toward the development of a diagnostic test for HIE at birth based on an infant’s metabolic profile.

The elevated amino acid and acyl-carnitine levels observed in our cohort of infants with HIE are consistent with the known pathophysiology of systemic hypoxic trauma. Disruptions in oxidative metabolism and the switch to anaerobic metabolism associated with HIE lead to mitochondrial membrane depolarization, intracellular accumulation of Ca^2+^ and extracellular build-up of excitatory amino acids^16–21^. The resulting metabolic acidosis is measurable, and others have reported alterations in amino acid and acyl-carnitine profiles similar to those described here^16,17,22–26^. Our finding that tyrosine was the screening analyte most strongly associated with HIE in our cohort has also been previously observed^25^. In one study, while acyl-carnitines levels were elevated in cases of HIE and more mild asphyxia, amino acid levels were increased only among infants with HIE. Such findings suggest that alterations in amino acid and acyl-carnitine levels may be indicative of the severity of brain injury^4,17^.

Additionally, our group has observed similar variations in the newborn metabolic profiles of infants with neonatal sepsis, among whom amino acids and acyl-carnitines were also most strongly associated with diagnosis^27^. Tyrosine was strongly associated with a diagnosis of neonatal sepsis among term infants, as it was with cases of HIE. However, tyrosine ratios were uniquely important in identifying infants with HIE, and tyrosine was generally less strongly associated with neonatal sepsis among preterm infants. The observed similarities in alterations of newborn metabolic profiles between cases of sepsis and HIE may suggest a generic pattern of catabolic stress experienced by the infant rather than a specific pathologic condition. As our cohort was limited to those infants who were born at term, further work to understand the association between catabolic stress and adverse neonatal outcomes would be beneficial to identifying underlying illness in term neonates not otherwise suspected of being unwell.

Given the narrow window during which therapeutic hypothermia should be initiated for optimal outcomes (~6 hours)^28–31^, and the fact that newborn screening heel prick samples are not typically collected until at least 24 hours after birth, further research exploring the possibility of sample collection immediately after birth is warranted. Previous research has indicated that cord blood may have particular value in determining the severity of HIE^17^, information that could be important in clinical decision-making and determining long-term prognosis. As cord blood samples can be collected within the first few minutes after delivery, examination of analytes in cord blood samples may represent a unique opportunity for identifying HIE within a clinically useful timeframe^17^. As cord blood pH is already measured for the purpose of HIE diagnosis, the collection of cord blood for additional metabolic testing should not be disruptive to clinical processes. Inclusion of routinely collected cord blood pH data could also serve to strengthen future models. Although our models do not address HIE severity or infant clinical outcomes, other investigators have sought to derive such algorithms by alternative methods^5^ including amplitude-integrated electroencephalography^32–34^, urinalysis^35,36^, and clinical signs in the first few hours after birth^37^. Future studies should investigate the added benefit of incorporating routinely measured metabolic markers into these other models.

A strength of our study was the size of the cohort, which allowed us to examine the newborn screening profiles of over 700,000 term infants and provided considerable statistical power to detect associations between individual analytes and HIE. A limitation of our study was the low sensitivity of diagnostic codes for identifying all cases of HIE in the hospitalization database, which may have resulted in some level of measurement error. All cases of HIE, including the most mild cases identified using the Sarnat staging criteria, would have been included in our cases, and previous research has indicated the ICD codes have many limitations in identifying cases of birth asphyxia^38,39^. However, we expect the impact of any such non-differential misclassification as a result of false-negative classification would have diluted any associations we observed. In order to mitigate this issue, we excluded preterm infants, as has been recommended in previous research due to the challenges of diagnosing HIE among this population as a result of coincident conditions^11,40^.

Further limitations stem from potential issues of confounding and under-reporting of other potentially important variables. Our analysis revealed statistically significant differences in several clinical variables between infants with and without HIE, including timing of bloodspot collection and use of parenteral nutrition. In order to limit the risk of confounding as a result of these differences, we controlled for all of these baseline variables in our analyses. Total parenteral nutrition (TPN) was reported among only 6% of infants with HIE in our cohort despite TPN being generally recommended for children who undergo therapeutic cooling^41^. This suggests that some instances of TPN may not have been recorded in the database. We were also unable to ascertain information regarding which infants received treatment. Infants with HIE are likely to have undergone therapeutic cooling within six hours after birth, a therapy that is both available and recommended as the standard of care for term infants with HIE^30,42–44^. It is unknown how many infants received therapy and what impact it may have had on their newborn screening profiles, but given the standard use of this treatment for HIE, it is likely to have been used in all cases that were not too mild or too severe to merit treatment. Further research should be undertaken to explore how infant cooling or other treatment may alter newborn screening analyte values. Lastly, because we excluded infants who had not undergone newborn screening, our sample is limited to infants who survived the first few days after birth, as newborn screening samples are generally collected 24–72 hours after birth. Thus, we may have missed those infants with the most severe cases of HIE who did not survive beyond this timeframe. We are unable to determine whether the metabolic profiles of these infants were different from those of the infants who survived.

Our study demonstrates proof-of-concept that metabolic markers have the potential to improve the predictive power of diagnostic and prognostic testing for HIE. The findings of our study support those from other related studies that infants with HIE exhibit distinct metabolic profiles consistent with the known pathophysiology of this condition. These results indicate a future potential to develop a test that uses routinely collected data to identify infants with HIE in a time period that would permit determination of appropriate interventions. As investigators seek to validate reliable, feasible and cost-effective tools, further research should examine the feasibility of using cord blood-derived newborn screening profiles immediately after birth for earlier detection of cases, risk stratification and guiding treatment. Importantly, we suggest that future work should explore the utility of newborn screening data to develop algorithms that might predict prognosis of long-term outcomes after HIE.

## Methods

This study was conducted at the ICES, a non-profit research organization with Prescribed Entity status under provincial privacy legislation. This study was approved by the Ottawa Health Science Network Research Ethics Board, the Children’s Hospital of Eastern Ontario Research Ethics Board, and the ICES Privacy Office. All study methods were conducted in accordance with the relevant guidelines and regulations set out by these institutions. The dataset from this study is held securely in coded form at ICES. While data sharing agreements prohibit ICES from making the dataset publicly available, access may be granted to those who meet pre-specified criteria for confidential access, available at www.ices.on.ca/DAS. The full dataset creation plan is available from the authors upon request.

### Study design, population and data sources

We carried out a population-based retrospective cohort study of all term (i.e., ≥37 weeks’ gestational age) live births in Ontario, Canada between January 1^st^, 2010 and December 30^th^, 2015. Data were available for infants born as early as 2006, but due to our interest in the role of fetal hemoglobin in predicting HIE and the fact that hemoglobin was not included in newborn screening in Ontario until 2010, we restricted our study cohort to infants born after January 1, 2010. Eligible infants were identified through screening records from Newborn Screening Ontario, a provincial program that screens all newborns for rare conditions using a panel of screening analytes obtained via heel prick blood spot. Blood spot samples are collected on filter paper and sent to Newborn Screening Ontario for screening for 29 rare, inherited conditions. All newborn screening samples in Ontario are analyzed in one central laboratory using standardized biochemical assays. The 49 individual analytes used in our study (Table S1) included acyl-carnitines (n = 31), amino acids (n = 12), relative fetal-to-adult hemoglobin level (n = 1), endocrine markers (n = 2), and enzymes (n = 3). Most analytes are measured by flow-injection tandem mass spectrometry. The exceptions are 17-hydroxyprogesterone (17OHP) and thyroid-stimulating hormone (TSH), immunoreactive trypsinogen (IRT) which are measured using a fluorescent immunoassay (GSP, Perkin Elmer, Waltham, MA); biotinidase, galactose-1- phosphate uridyltransferase (GALT) activity measured using a colorimetric enzyme assay (Spotchek Pro; Astoria-Pacific, Inc, Clackamas, OR); and hemoglobinopathies detected using ion-exchange chromatography (Variant, Bio-Rad Laboratories (Canada) Ltd., Montreal, QC). Additional information about laboratory analysis methods can be found in the Supplementary Materials.

Infants were excluded if they screened positive for any disorders in the screening panel in order to minimize outlier analyte values (n = 11,863), if their sample quality was unsatisfactory (n = 12,665), and if they were transfused before blood spot collection (n = 4,693), as donor blood may impact certain analyte levels. We also excluded those without continuous eligibility for provincial healthcare benefits between birth and the end of the neonatal period (i.e., up to 28 days of age; n = 6,215). Infants who died during this time were not excluded unless they were ineligible for healthcare benefits at the time of their death. A flowchart of sample exclusions is included as Fig. 1. To ascertain diagnoses of HIE during the neonatal period, we linked the study cohort to hospitalization records from the Canadian Institute for Health Information’s Discharge Abstract Database (DAD). These datasets were linked using unique encoded identifiers and analysed at the ICES.

A small Canadian validation study of these ICD-10 diagnostic codes from the DAD against a clinical perinatal database found sensitivity and specificity values of 38.9% and 99.9%, respectively (Reproductive Care Program of Nova Scotia, unpublished data, 2016). In order to compensate for this low sensitivity and for known temporal changes in the codes used in the DAD^38^ to identify cases of HIE, neonatal HIE was defined as having any of the following International Classification of Disease 10^th^ Revision (ICD-10) diagnostic codes recorded during any hospitalization initiated within the neonatal period: P916 (hypoxic ischaemic encephalopathy of newborn), P20 (fetal asphyxia), P21 (birth asphyxia), G931 (anoxic brain damage, not elsewhere classified).

### Variable measurement

We assessed the correlation between HIE and (1) each analyte and (2) each analyte ratio (a complete list of analytes and correlations can be found in Supplementary Table S2). To minimize the impact of extreme values, outliers (i.e., those below the 0.001^st^ percentile or above the 99.999^th^ percentile) were replaced with analyte value at those percentile levels. Analytes were standardized to the standard normal distribution to render a unit change in each analyte comparable regardless of different measurement scales. This standardization was conducted by calendar week of birth of the screened infant to minimize variation due to external factors.

### Model development

We then developed prediction models using multivariable logistic regression with HIE as the binary dependent variable to determine whether the addition of screening analytes improved the identification of HIE cases compared to clinical variables alone. We began with a baseline model (Model 1) containing only clinical factors (sex, birthweight, plurality, gestational age, age at collection, and feeding status), to which we then added categories of analytes, in an order based on pathophysiological parameters as well as mechanism of measurement, as we have done in previous studies^27,45^. We started by adding a variable for relative fetal-to-adult hemoglobin (Hb) levels (Model 2), which is measured by high performance liquid chromatography and is a marker of oxygen delivery to tissues. In Model 3, we added TSH and 17-OHP, both of which are markers of endocrine function measurable using non-mass spectrometry methods. Finally, in Model 4, we added the remaining analytes and analyte ratios comprised of acyl-carnitines and amino acids which are measured using tandem mass spectrometry methods. Due to the large number of available variables in the latter category, we added variables according to rank-order based on Spearman’s correlation until the maximum number of parameters was reached, based on prior consideration of the number of cases. Restricted cubic splines were used to model the top five ranked analytes/analyte ratios in order to capture non-linear associations. The number of analytes to model using splines was made after considering the ranked partial Spearman’s correlations.

### Model discrimination

To assess model discrimination, we used the area under the receiver operating characteristic curve (AUC) and the Integrated Discrimination Improvement (IDI) to quantify the impact of additional groups of analytes on the average sensitivity of the preceding nested model. We used the Akaike Information Criterion (AIC) to assess each model’s incremental improvements in fit, which accounts for the increasing model size to prevent over-fitting. We also used the Net Reclassification Improvement (NRI) to quantify the change in predicted values compared to the preceding nested model. Analyses were conducted with SAS version 9.4 (SAS Institute, Inc., Cary, North Carolina) and with R/R Studio using the RMS and HMISC packages.

## Supplementary information

Supplementary Materials

## Data Availability

The dataset from this study is held securely in coded form at ICES. While data sharing agreements prohibit ICES from making the dataset publicly available, access may be granted to those who meet prespecified criteria for confidential access, available at www.ices.on.ca/DAS. The full dataset creation plan and underlying analytic code are available from the authors upon request, understanding that the computer programs may rely upon coding templates or macros that are unique to ICES and are, therefore, either inaccessible or may require modification.
