# Supplementary material for: Association between newborn screening analytes and hypoxic ischemic encephalopathy
Source: Sci Rep. 2019 Oct 31;9:15704. doi: 10.1038/s41598-019-51919-x (PMC6823438; doi:10.1038/s41598-019-51919-x)
Supplement: Supplementary file 1 — Supplementary Materials [file 41598_2019_51919_MOESM1_ESM.pdf]

Association between newborn screening analytes and hypoxic ischemic encephalopathy

Wilson LA, Fell DB, Hawken S, Wong CA, Murphy MSQ, Little J, Potter BK, Walker M, Lacaze-Masmonteil T, Juul S, Chakraborty P, Wilson K

Supplementary Materials

Supplemental information on newborn screening laboratory methods

Amino acids, acylcarnitines and succinylacetone (SUAC) are extracted from 3.2 mm punches from dried blood spots, in 96 well plates, using 90µL methanol:water containing stable isotope internal standards and then butylated using 50µL Butanol-HCl at 65°C for 30 min. Residual blood spots are re-extracted using 100µL of an acidic (50 µL 99% Formic Acid) hydrazine-hydrate solution with labelled internal standard to form a hydrazine derivative of SUAC[1]. Extracts are dried down, reconstituted in 75µL of acetonitrile/water (4:1 v/v) and combined; 10µL are injected and analyzed using Flow injection MS/MS with a run time of 1.7 min using positive electrospray ionization on a Waters TQ Detector utilizing a combination of Multiple Reaction Monitoring, Neutral Loss of 102 Da and Precursor ion (Parents of 85 Da) scanning. Data acquisition, processing and quantification were performed with the NeoLynx™ Application Manager, as part of the MassLynx v4.1 software, calculating simple peak intensity ratios and thus analyte concentrations.

1. Allard P, Grenier A, Korson M and Zytkovicz T; Newborn screening for hepatorenal tyrosinemia by tandem mass spectrometry: analysis of succinylacetone extracted from dried blood spots; Clinical Biochemistry 2004;37,1010-1015

Table S1. Individual newborn screening analytes used as independent variables in model development

Acyl-carnitines (n=31)	C0 (carnitine)<br>C2 (acetylcarnitine)<br>C3 (propionylcarnitine)<br>C4 (butyrylcarnitine)<br>C5 (valerylcarnitine)<br>C5:1 (Tiglylcarnitine)<br>C6 (hexanoylcarnitine)<br>C8 (octanoylcarnitine)<br>C8:1 (octenoylcarnitine)<br>C10 (decanoylcarnitine)<br>C10:1 (decenoylcarnitine)<br>C12 (dodecanoylcarnitine)<br>C12:1 (dodecenoylcarnitine)<br>C14 (tetradecanoylcarnitine)<br>C14:1 (tetradecenoyl carnitine)<br>C14:2 (tetradecadienylcarnitine)	C16 (hexadecanoylcarnitine)<br>C18 (octadecanoylcarnitine)<br>C18:1 (octadecenoylcarnitine)<br>C18:2 (octadecadienylcarnitine)<br>C4OH (hydroxybutyrylcarnitine)<br>C5DC (glutarylcarnitine)<br>C5OH (hydroxyvalerylcarnitine)<br>C6DC (methylglutarylcarnitine)<br>C14:OH (3-hydroxytetradecanoylcarnitine)<br>C16:OH (hydroxyhexadecanoylcarnitine)<br>C16:1OH (hydroxyhexadecenoylcarnitine)<br>C18OH (3-hydroxystearoylcarnitine)<br>C18:1OH (hydroxyoctadecenoylcarnitine)<br>C3DC (malonylcarnitine)<br>C4DC (methylmalonylcarnitine)
Amino acids and related markers (n=12)	Arginine<br>Phenylalanine<br>Alanine<br>Leucine<br>Ornithine<br>Citrulline	Tyrosine<br>Glycine<br>Argininosuccinate<br>Methionine<br>Valine<br>Succinylacetone
Relative fetal-to-adult hemoglobin (Hb) level (n=1)	Fetal hemoglobin (HbF+HbF1) / (Fetal hemoglobin (HbF+HbF1) + Adult hemoglobin (HbA))	
Endocrine markers (n=2)	17-hydroxyprogesterone (17-OHP)<br>Thyroid stimulating hormone (TSH)	
Enzyme markers (n=3)	Biotinidase (BIOT)<br>Galactose-1-Phosphate Uridyltransferase (GALT)<br>Immunotripsinogen (IRT)	

Table S2. Spearman's Rank Correlation between Analytes/Analyte Ratios and HIE

Analyte/Analyte Ratio	Min Value	Max Value	Partial R
tyr	-2.874	14.68	0.002
tyr_met	-2.656	18.79	0.002
ala_tyr	-2.047	53.19	0.002
tyr_val	-2.706	45.85	0.002
tyr_gly	-2.553	21.23	0.002
c12_1	-2.387	16.28	0.001
tyr_hgb_ratio	-2.901	33.80	0.001
phe_tyr	-2.426	53.46	0.001
leu_tyr	-2.523	53.44	0.001
c0_tyr	-1.687	52.70	0.001
c5_tyr	-1.785	52.16	0.001
c8_1_tyr	-1.690	53.38	0.001
orn_tyr	-2.267	52.96	0.001
tyr_biot	-2.315	51.18	0.001
c18_2_tyr	-1.377	52.21	0.001
tyr_irt	-1.689	28.37	0.001
c2_tyr	-1.904	53.31	0.001
tyr_suac	-2.405	31.39	0.001
c10_1_tyr	-1.906	53.13	0.001
c18_1_tyr	-2.118	53.14	0.001
cit_tyr	-2.176	53.40	0.001
c0_c12	-1.844	32.29	0.001
c8_1_c12_1	-1.929	33.25	0.001
c12_1_ala	-2.264	24.83	0.001
arg_tyr	-1.645	53.51	0.001
c12_ala	-2.109	20.24	0.001
c4dc_tyr	-1.881	53.55	0.001
c0_c12_1	-2.221	31.59	0.001
c8_tyr	-1.918	53.45	0.001
c14_1_ala	-1.854	12.39	0.001
c0_c14_1	-1.678	30.00	0.001
tyr_galt	-2.240	50.18	0.001
c0_c14	-2.332	45.11	0.001
c5oh_tyr	-2.023	53.44	0.001
c10_1_c12_1	-1.941	39.82	0.001
c6_tyr	-1.886	52.98	0.001

c2_c12	-2.405	39.58	0.001
c4_tyr	-1.630	53.21	0.001
c8_1_c12	-2.303	38.56	0.001
c8_c12_1	-2.096	32.99	0.001
c8_1_c14_1	-2.410	37.41	0.001
c12_1_val	-2.274	40.28	0.001
c6dc_ala	-2.139	24.12	0.001
c14_2_tyr	-1.771	53.17	0.001
c5_c12_1	-1.654	35.66	0.001
c3_tyr	-1.771	52.82	0.001
c12_1_met	-2.312	20.20	0.001
c18_tyr	-1.972	53.36	0.001
c12_1_irt	-1.554	27.92	0.001
c14_1_val	-2.033	43.47	0.001
c5_c14_1	-1.528	31.72	0.001
c3dc_ala	-2.544	13.87	0.001
c16_tyr	-2.097	53.49	0.001
c12_1_c18_2	-2.036	39.52	0.001
c12_1_gly	-1.859	19.77	0.001
c2_c12_1	-2.576	30.47	0.001
c2_c14_1	-2.072	33.28	0.001
c12_c18_2	-1.789	32.67	0.001
c14_1_c18_2	-1.643	37.76	0.001
c6dc_c8_1	-1.713	33.57	0.001
c5_c12	-1.496	34.70	0.001
c3dc_tyr	-2.327	53.47	0.001
c8_1_c10	-1.968	33.61	0.001
c5dc_ala	-2.307	13.42	0.001
c0_c16	-1.957	35.11	0.001
c14_ala	-2.338	13.82	0.001
c12_val	-2.249	45.28	0.001
tyr_asa	-2.254	50.66	0.001
c8_1_c14oh	-1.280	26.81	0.001
c3dc_c8_1	-1.860	28.54	0.001
c14_1_met	-2.053	12.84	0.001
c12_1_phe	-2.525	18.15	0.001
c12_met	-2.316	19.26	0.001
c14oh_ala	-1.844	33.01	0.001
c4oh_ala	-2.159	12.78	0.001

c5_1_tyr	-1.664	52.70	0.001
c10_1_c14_1	-1.809	43.43	0.001
c0_c4oh	-1.872	40.45	0.001
c5dc_c8_1	-1.766	26.71	0.001
c12_irt	-1.560	27.39	0.001
c10_ala	-1.948	18.51	0.001
c10_1_c12	-1.978	33.13	0.001
c14_1_gly	-2.192	11.03	0.001
c14_1_irt	-1.530	27.46	0.001
c12_gly	-2.470	14.66	0.001
c12_1_hgb_ratio	-2.177	28.76	0.001
